# Supplementary material for: Are carbon emissions trading and green financial instruments synergistic? -Comprehensive quantitative research based on content analysis
Source: PLoS One. 2024 Mar 7;19(3):e0298601. doi: 10.1371/journal.pone.0298601 (PMC10919601; doi:10.1371/journal.pone.0298601)
Supplement: S1 Data — (ZIP) [file pone.0298601.s001.zip › Data/地级市Z_F工作报告.zip!/║■─╧/┬a╡╫╩╨/└┤╫╘╣1⁄2╓┌║┼í╛┬φ┐╦╩2╛▌═°í┐_2019.docx]

各位代表：

现在，我代表市人民政府，向大会作工作报告，请予审查，并请各位政协委员和列席人员提出意见。

一、2018年工作回顾

过去一年，全市上下坚持以习近平新时代中国特色社会主义思想为指导，深入贯彻党的十九大精神，认真落实党中央国务院、省委省政府和市委的决策部署，按照高质量发展要求，大力实施创新引领开放崛起战略，加速转型，奋力赶超，完成了全年各项目标任务。

主要经济指标稳中有进。地区生产总值增长8%（预计数，下同），其中一二三产业分别增长3.5%、7.7%、9.5%；固定资产投资增长10%；规模工业增加值增长7.5%；社会消费品零售总额增长10.5%；一般公共预算收入增长13.98%；城乡居民人均可支配收入分别增长8.5%、11%；居民消费价格指数上涨2%；节能降耗超额完成省定任务。

十件民生实事总体完成。完成年度任务的有8件，驻村服务全科医生或助理全科医生达到1227名；79个易地扶贫集中

安置项目基本完成，搬迁24341人；垃圾分类试点村达到946个；义务教育班额基本控制在65人以下；1900个自然村通水泥（沥青）路，建设里程1691公里；湘江资江娄底流域截污和水体治理完成12公里；山塘清淤完成4800口；中心城区小游园建成9个。没完成时序进度的有2件，娄底大道冷新段建设，完成投资计划的97%；背街小巷第一期改造，完成投资计划的95%。

七个“三年行动计划”基本完成。工业提振实现规模工业增加值年均增长7.6%，规模工业企业达到857户。文化旅游及健康养老产业完成投资360亿元，年均增长35%，总收入达到1395亿元，年均增长24%。中心城区提质扩容完成总投资247.6亿元，娄星南路、吉星路、甘桂路等12条“断头路”全部打通。脱贫攻坚减少建档立卡贫困人口30.62万，退出贫困村397个，易地扶贫搬迁68544人。财源培植实现一般公共预算收入年均增长11.33%。天蓝水净地绿行动初见成效，城区细颗粒物年均浓度下降44%，县以上城镇污水处理率达到95.33%，中心城区建成区绿地率达到35.48%。综合交通大为提升，二广、娄衡、益娄高速公路建成通车，娄底大道娄涟段等10个国省干线公路项目竣工，行政村全部开通客运班线。七个“三年行动计划”的实施，为决胜全面小康、完成“十三五”规划奠定了坚实基础。

2018年，我们坚决守底线，奋力争上游，在七个方面取得了好的成绩：

（一）三大攻坚战扎实推进。全面清理核实政府性债务，调减政府投资项目141个，压减投资483.72亿元，清理规范PPP及政府购买服务项目59个，压减投资387.47亿元，政府性债务风险总体可控。采取“6项措施”，加快推进民间融资风险处置，集中办理了一批大案要案，平稳化解了九龙集团“6·30”风险。建立地方金融风险监测预警联动机制，14家省级金融机构与我市签订了战略合作协议，授信2365亿元。

深入推进脱贫攻坚“七大行动”和“七大扶贫工程”，全年减少建档立卡贫困人口11.34万，223个贫困村退出，双峰县可以脱贫摘帽。狠抓扶贫领域作风建设，召回不合格工作队2个，追究派出单位领导责任14人。推进市委“四个意见”落地见效，激发群众内生动力，16名村支部书记被评为“脱贫致富带头人”，29名脱贫对象被评为“自强自立先进个人”。

抓实中央、省环保督察反馈问题整改，积极配合开展“回头看”，中央环保督察交办的245个信访件全部办结，反馈的9个问题5个办结销号，其余4个正在加快整改。有效实施“湘江保护和治理第二个三年行动计划”“锡矿山区域环境综合治理三年行动计划”，成功申报全国第三批山水林田湖草生态修复试点工程。市级饮用水水源地整治全面完成，畜禽规模养殖污染防治、城市黑臭水体整治、土壤与重金属污染治理、采（碎）石场规范化整治全面推进。中心城区空气质量优良天数比例为90%，全市集中饮用水水源地水质达到或优于Ⅲ类。

（二）项目建设成效明显。扎实开展“产业项目建设年”活动和“项目建设大比拼”，全年推进各类项目1670个，其中重大产业项目328个，完成投资445亿元。列入省“五个100”的项目13个，完成投资41.8亿元，三迅新能源高能量动力电池产品研发项目获评省重大科技创新优秀项目。先后在长沙、北京、杭州开展了互联网+装配式建筑、军民融合、特色小城镇等产业链专题招商，签约项目61个，总投资268.77亿元。园区项目集聚能力增强，园区规模工业增加值增长8.8%，占全部规模工业的比重达到70.2%，娄底经开区获批全国大中小企业融通型特色载体，娄星工业集中区成功创建湖南省军民融合产业示范园区、创新创业示范基地，新化县经开区获批省级高新区。娄底高新区创建国家级高新区有序推进。

（三）转型发展步伐加快。株潭娄国家产业转型升级示范区建设初见成效，围绕建设“钢铁新城”、打造新材料新能源基地，重点推进了12条产业链建设。薄板深加工及汽车零部件千亿产业链基本完成布局，已规划入园项目4个，华安钢宝利二期竣工投产；新能源动力电池及储能产业链已有电将军第一条生产线等8个项目投产，3个项目在建；军民融合产业链已聚集28家企业，其中15家企业获得军工证；装配式建筑等其它9条产业链加快布局推进。农业与二三产业加快融合，新增规模以上农产品加工企业49家、休闲农业企业492家，建成乡、村电子商务服务站点1829个，农产品加工销售收入、经营收入分别增长16%、17%。新增中国驰名商标3个、国家地理标志登记注册3个、农业产业化国家重点龙头企业1家，3个产品获第十六届中国国际农产品交易会金奖，“月光红”获评中国“茶王”。双峰县被授予“中国农业机械之乡”称号，涟源市桥头河镇入选全国现代农业强镇。商贸物流、文化旅游、健康养老等现代服务业来势较好，娄底旅游成功纳入湖南旅游中部崛起战略，旅游收入和人数分别增长18%、19.3%。互联网经济、总部经济、赛事经济等新业态新模式加快发展，万宝移动互联网产业园已吸引陌陌、谷歌等企业70余家，新化县上市小镇已有34家准上市企业入驻。科技创新力度加大，财政科技支出增长98%，引进科技创新人才8名，新增国家级创新研发平台2家、院士工作站2家、产业技术联盟10家。

（四）改革开放继续深化。有力有序推进35大类106项改革举措。“放管服”改革梳理“只跑一次”事项1007项，完成91.9%。“互联网+政务服务”一体化平台上线运行，信息共享交换平台投入使用，我市荣获“2018中国智慧城市创新奖”。全面实施企业登记“37证合一”，实行“一网通办”“一窗核发”，企业登记压缩至8.5个工作日，不动产登记压缩至5个工作日。农村土地承包经营权确权登记颁证全面完成，农村集体产权制度改革全面推进。坚持招大引强，签约落户500强企业项目9个，总投资179亿元。成功举办第九届“湘博会”和第五届娄商大会，签约项目50个，总投资351.5亿元。全年实际利用外资5.5亿美元，增长19%；内联引资700亿元，增长15.4%。海关总署批准设立娄底海关，获批外贸综合服务中心5家，新增外贸进出口资质企业83家，全市进出口总额增长25%，冷水江市获评全省发展开放型经济优秀县市。

（五）城乡面貌大幅改观。中心城区创文管卫工作制度化常态化，交通秩序、超限超载、控违拆违等专项整治成效明显。城市“双修”有力推进，完成投资19.2亿元，5个背街小巷片区、9条城市道路（18.5公里）完成提质改造，12条城市道路加快建设，一批群众反映强烈的城市基础设施得到改善，城区品质和形象得到提升。秀美县城、特色小城镇、美丽乡村建设深入推进，“乡村振兴15条”初见成效，娄星区、冷水江市获评“全省美丽乡村建设工作先进县市区”，双峰县三塘铺镇入选“中国最具魅力乡镇”，新化县土坪村获评“中国美丽休闲乡村”。完成干线公路改造114公里、农村公路提质改造303公里，娄底大道双峰段基本完工，龙琅高速公路建设进展顺利。

（六）社会民生全面进步。全市民生支出占到一般公共预算支出的74.8％，省定重点民生实事项目全面完成。城镇新增就业3.84万人，新增农村劳动力转移就业3.96万人，城镇登记失业率为3%。补充教师2641名，新增学位31820个，双峰县、涟源市被认定为全国义务教育发展基本均衡县（市）。完成农村危房改造12951户、保障性住房 18211套，中心城区老旧小区改造2856户。城乡低保月人均救助水平分别提高7.04%、36.7%。公立医院药占比降至31.34%。建成村级综合文化服务中心1391个，组建新时代乡村业余文艺宣传队87支，6家文化企业荣获第九届西部文博会“优秀创意产品奖”。深入开展安全生产“十大战役”，强化“打非治违”“强执法防事故”，事故起数、死亡人数分别下降6%、9%，全年没发生较大及以上安全生产事故，获评全省安全生产先进市。重要工业产品、食品抽检合格率分别为97.3%、98.71%。非洲猪瘟疫情得到有效防控。深入推进扫黑除恶专项斗争，摧毁黑社会性质犯罪组织5个，打掉恶势力团伙59个。创建全国禁毒示范城市成功入榜，1235个行政村建立了治安队，无上访乡镇（街道）达到23个、无上访村（社区）达到676个。

（七）自身建设不断加强。坚持和加强党的全面领导，深入推进“两学一做”学习教育常态化制度化，落实市政府党组理论学习中心组学习制度，切实提高推动高质量发展的能力和水平。主动接受人大及其常委会的法律监督、工作监督，3位副市长和8个政府工作部门接受了人大评议。自觉接受政协民主监督，及时通报政府工作情况。全年办结人大代表议案1件、建议116件、政协委员提案162件，所提问题得到解决或基本解决的148件、列入计划逐步解决的118件、暂时难以解决做出解释说明的13件。办理市长信箱来信1010件、红网留言521件，接听12345市民来电17009件、登记事项11789件，办结率、满意率分别为98.5%、98.3%。推进立法、普法、执法工作全面加强，《孙水河保护条例》正式实施，《娄底市湄江风景名胜区条例》已经省人大常委会审查批准，《仙女寨生态公园保护条例》完成第一次审议，在全省首次组织政府行政执法监督员对环保系统开展专项检查，推动行政机关负责人出庭应诉45次。出台了市政府党组关于加强党风廉政建设的十条规定，坚持“零容忍”惩治腐败。加强审计监督，查出违法违纪金额8.8亿元，经审计上缴财政4.2亿元，移送案件线索43起。严格落实中央八项规定及实施细则，整合减少各类考核评比，有效精简会议和文件，清理整改超标办公用房3.91万平方米，党政机关、市属国有企业公车改革顺利完成。

国防动员和后备力量建设全面加强，军民融合深度发展。统计、消防、人防、地震、气象、移民、供销、公共资源交易、档案、机要、保密、方志、对台、贸促、外事侨务、港澳、民族宗教、工商联、工会、共青团、妇联、老龄、关工委、老促会、文联、科协、侨联、社科联、红十字会、慈善、残联、计生协等各项工作，均取得了新的进步。

一年来，我们坚持工作高标准、严要求，涌现出了不少特色和亮点。一是聚精会神搞建设、抓发展，主要经济指标完成较好，13项主要经济指标中，10项指标增速进入全省前列，赶超态势得到巩固。二是突出项目建设主抓手，引进新上了湖南利钢、益盛新材、三一路机、博盛新材、杭萧钢构、新印科技等一批大项目、好项目，新增规模以上企业363家，其中规模工业企业150家、高新技术企业40家，为发展增强了后劲、提供了支撑。三是以质量和效益为核心，加快产业转型与结构优化，三次产业结构调整为10:46.4:43.6，第三产业贡献率达到46.9%；产业投资增长26.2%，占全部投资的60.9%，其中工业投资增长25.7%，工业技改投资增长31.1%，高新技术投资增长137.7%；纳税过亿元的企业从上年的6家增加到13家，全市税收占比达到68%，高质量发展的态势初步形成。四是抓重点、补短板、强弱项，全力打好了三大攻坚战，守住了安全生产、信访维稳等工作底线，解决了民生领域的一些突出问题，人民群众有了更多获得感。五是创造性地开展了一些工作，“村级小微权力+互联网+监督”改革探索被中纪委、新华社推介，新化县油溪桥村积分制管理被评为全国乡村治理十大经典案例之一，成立全省首个区块链产业园区、发放了全国首张不动产区块链电子凭证，河长制工作获全国先进，冷水江市进入全国小康百强县市行列，等等。这些特色和亮点工作，体现了娄底智慧，增强了娄底自信，铸就了娄底荣誉！

各位代表！

刚刚过去的2018年，是改革开放40周年，是贯彻落实党的十九大精神的第一年，也是我市推进七个“三年行动计划”的收官之年。40年来，全市人民高举开放大旗，锐意改革，开拓创新，砥砺奋进，推动了娄底的快速发展，成就了“湘中巨变”。尤其是近几年，全市上下主动扛起全面建成小康社会的历史重任，紧紧围绕建设富饶美丽健康祥和新娄底的目标，履职尽责，攻坚克难，争先创优，用实际行动向改革开放40周年，交出了一份娄底答卷，从2016年的“合格答卷”，到2017年的“优异答卷”，再到2018年的“好”成绩单。娄底大地呈现出迎难而上、奋发向上、蒸蒸日上的新气象，续写了稳中有进、稳定向好、稳步提质的新篇章。我们可以自豪地说，今天的娄底，已不再“垫底”，正在稳步“上楼”！

各位代表！

2018年各项成绩的取得，是市委总揽全局、坚强领导的结果，是市人大、市政协有力监督、鼎力支持的结果，是各级各部门迎难而上、团结奋进的结果，凝聚了全市人民和社会各界的智慧和力量，倾注了广大干部职工的心血和汗水。在此，我谨代表市人民政府，向全市人民，向各民主党派、工商联、人民团体，向驻娄人民解放军和武警部队指战员、政法干警，向中央、省驻娄单位，向所有关心支持我市发展的各界人士，表示衷心的感谢并致以崇高的敬意！

与此同时，我们也深切感受到，经济社会发展中还存在一些短板和弱项，工作推进中还存在一些不足和问题。一是部分经济指标增速虽然在全省排名靠前，但离年初目标有差距，经济发展还面临较大的下行压力。二是经济总量不大、结构不优的状况尚未根本转变，新项目、大项目、好项目还不够多。三是三大攻坚战的任务还很重，基础设施、公共服务、生态治理、社会民生等领域的欠账较多，区域协调发展难度较大。四是审批难、征拆难、融资难、推进难等问题较为突出，营商环境不够优，安全生产、信访维稳、基层治理压力较大，阻碍发展的制约因素依然存在。五是少数干部思想观念保守、担当意识不强、工作作风不实，抓经济工作、项目建设的精力不够集中，与高质量发展的要求不适应，等等。我们将始终坚持问题导向，一个一个地加以解决。

二、2019年目标任务和工作重点

2019年，是新中国成立70周年，是全面建成小康社会关键之年，是娄底建市20周年。面对复杂严峻的外部环境和经济下行压力，我们一定要坚定信心，把握主动，危中求机，化危为机，坚定不移地办好自己的事情，切实将中央、省委和市委的决策部署落细落实。

2019年政府工作的总体要求是：以习近平新时代中国特色社会主义思想为指导，认真贯彻习近平总书记关于湖南工作的重要指示精神，坚持稳中求进工作总基调，贯彻新发展理念，坚持以供给侧结构性改革为主线，深化市场化改革，扩大高水平开放，实施创新引领开放崛起战略，继续打好三大攻坚战，继续开展项目建设大比拼，进一步稳就业、稳金融、稳外贸、稳外资、稳投资、稳预期，推动高质量发展，实现加速转型、奋力赶超，保持经济持续健康发展和社会大局稳定，为全面建成小康社会收官打下决定性基础，以优异成绩庆祝中华人民共和国成立70周年。

2019年的主要预期目标是：地区生产总值增长7.7%以上，其中一二三产业分别增长3%、7.3%、9.5%以上；一般公共预算收入增长8%以上；固定资产投资增长10.5%以上，其中产业投资增长13%以上，工业投资增长15%以上，高新技术投资增长20%以上，工业技改投资增长16%以上；研发投入增长25%以上；申报高新技术企业30家以上；城乡居民人均可支配收入分别增长8%、10.5%以上；居民消费价格涨幅3%左右；金融财政风险有效防控；节能降耗完成省定目标。

围绕上述目标，重点抓好六个方面的工作：

（一）继续打好三大攻坚战，补齐高质量发展短板

防范化解重大风险。加强政府债务限额管理，推动政府平台公司市场化转型，妥善化解债务存量，确保不增加债务总量。加强财政预算绩效管理，强化预算约束，提高使用效益。抓好“6项措施”落实，运用法治化、市场化手段，稳妥处置民间融资风险。健全完善金融风险防范处置机制，严厉打击逃废金融债务行为，加大互联网金融监管力度，积极创建金融生态良好城市和省级金融安全区，坚决守住不发生区域性金融风险底线。

大力推进精准脱贫。聚焦深度贫困村和特定贫困人群，重点解决好“两不愁三保障”问题，扎实推进产业、就业、教育、健康扶贫，以及易地扶贫搬迁、农村危房改造、兜底保障等工作，确保减少8万以上建档立卡贫困人口、90个以上贫困村脱贫出列、涟源市和新化县脱贫摘帽。贯彻落实好市委“四个意见”，加大驻村、结对、社会、企业联村帮扶力度，继续开展“五治行动”和“脱贫致富带头人”“自强自立先进个人”评比活动，形成更大脱贫合力。深入推进扶贫领域作风建设，加强资金监管，严惩扶贫领域形式主义、官僚主义、数字脱贫、虚假脱贫等突出问题，着力提升脱贫质量。开展脱贫对象回头看、回头查、回头帮，加大易地扶贫搬迁后续扶持力度，减少和防止贫困人口返贫，进一步巩固脱贫成果。

着力抓好污染防治。强化政治担当，不折不扣抓好中央环保督察“回头看”、省环保督察反馈问题整改落实，着力解决群众身边的突出环保问题。继续实施“湘江保护治理第二个三年行动计划”“锡矿山区域环境综合治理三年行动计划”，加强锡矿山地区、水府庙库区、采煤沉陷区、废弃矿山等重点区域集中治理，实施涟钢、冷钢环保达标提质行动，强力推进煤矿废水、煤矸石、采（碎）石场、石灰窑、粘土砖厂及城市规划区烧结砖厂等专项整治，全面完成“清四乱”专项整治任务。统筹山水林田湖草整体保护、系统修复、综合治理，加大黑臭水体治理、建筑垃圾处理力度，完成中心城区第一、二污水处理厂提标改造（扩建），推进乡镇污水处理厂建设和运营机制改革。进一步强化河长制责任落实，继续抓好农业面源污染防治，开展沿河排污口专项整治，持续开展零碳区域创建，建设孙水、涟水、资水和侧水生态长廊。推动资源综合循环利用，开展重点企业环保示范创建和重点用能企业“百千万”行动，推进国家第三批增量配电业务改革试点，加快“气化娄底”天然气管道建设，全面完成燃煤锅炉及经营性小煤炉淘汰整治任务。

（二）全力稳增长促转型，强化高质量发展支撑

推动工业提质升级。以国家产业转型升级示范区建设为总揽，加快建设“钢铁新城”。深度对接“中国制造2025”“湖南制造强省五年行动计划”，瞄准“湖南工业新兴优势产业链行动计划”，做大做强薄板深加工、新能源新材料、汽车及汽车零部件、生物医药、节能环保、电力安装等重点产业，全力推进薄板深加工产业园、新能源动力电池及储能产业园、光电子产业园、电力科技园、汽车产业配套基地等战略性新兴产业项目建设。全面开展中小企业“上云”行动，推动互联网、大数据、人工智能与实体经济深度融合，全面提升产业装备智能化水平。确保规模工业增加值增长7.6%以上、新增规模工业企业100家以上。

加快服务业创新发展。推进服务业提升“三大工程”，创建一批国、省级服务业集聚区，重点培育一批服务业龙头企业。加快通往主要景区公路建设，完善接待体系，打造以娄底城区为中心的1小时休闲度假旅游圈。推进曾国藩故居、紫鹊界梯田创建5A级景区，发展以锡矿山、涟钢为重点的工业旅游，支持新化县创建国家全域旅游示范区，确保旅游接待人次、旅游总收入两项指标均增长18%以上。做好湘军文化和梅山文化文章，推进“五名工程”，打造曾国藩家教文化、梅山傩戏等文化品牌。支持湘中国际物流园和诚通现代物流园做大做强，抓好各类专业市场培育，加快构建与高质量发展相匹配的现代物流体系，降低综合物流成本，提高物流效率。深入推进家政服务、文化体育、健康养老等消费提质提档，增强消费拉动能力，确保社会消费品零售总额增长10.5%以上。

培育壮大新兴优势产业。大力培育“小巨人”“独角兽”企业。做好顶层设计，制定扶持政策，做大做强移动互联网产业园，立足区块链、大数据，全力引进人工智能、量子信息、物联网、移动通信项目，推动数字经济快速发展。加强与异地商会联系，千方百计引进更多总部经济项目。加快足球新城、美食文化街建设，大力发展赛事、会展和夜游经济，培育新的经济增长点。

大力支持民营经济发展。进一步优化环境，任何政府部门都不得随意干扰企业，让企业安心生产、放手发展。引导民营企业讲正气、走正道，诚实守信，合法经营，回报社会。充分尊重民营企业家，构建亲清政商关系，弘扬优秀企业家精神，培育发展更多优质企业。全面落实中央减税降费等6个方面政策、湖南促进民营经济高质量发展的“25条意见”，确保民营企业应享尽享。全国其他地级以上城市出台的政策，只要符合娄底实际，没有禁止性、限制性条款，在娄底都可落地执行。

（三）大力抓项目优投资，增强高质量发展动能

加快项目推进。以省“五个100”、市“五个一批”项目为重点，继续开展“产业项目建设年”活动和“项目建设大比拼”，建立完善产业项目建设推进落实机制。推进市级重点建设项目218个，总投资1828亿元，年度计划投资375亿元，其中产业项目112个，总投资949亿元，年度计划投资188亿元；基础设施项目48个，总投资640亿元，年度计划投资103亿元；社会民生及生态环保项目40个，总投资239亿元，年度计划投资84亿元。抓好项目谋划，以现代制造业、新型城镇化为重点，紧盯乡村振兴、基础建设、生态环保、社会民生等重点领域，谋划重大项目500个左右，其中产业项目200个左右。

夯实园区载体。着力打造园区“725”升级版，即立足7个省级以上园区、建成200万平方米标准厂房、入驻500个规模以上企业。以娄底经开区为龙头，继续推进“一区多园”建设，建好“飞地园区”，引进“飞地产业”，实现园区政策共享、错位发展、优势互补，力争娄底高新区升格为国家级高新区。全面推行园区项目全程代办、每月联审制度，探索推进制造业项目“先建后验”“预审代办”，激发园区发展活力。推进产城融合，适当提高工业用地中商住用地比例，鼓励建设多层厂房，完善水电路气运等配套设施，增强园区承载能力，提升园区综合效益。确保园区规模工业增加值增长10%以上，园区工业占全市规模工业的比重达到72%。

强化要素保障。积极争取中央和省预算内资金、乡村振兴和三大攻坚战专项资金、国家小城镇发展基金，创新投融资体制机制，组建政府产业引导基金，完善融资担保体系，切实缓解融资难题。坚守耕地红线，加大废弃工矿用地、易地扶贫搬迁户老宅基地复垦力度，加强“空心村”治理，建立重点项目用地市级统筹机制，切实解决用地难、推进难等问题。实施“娄底人才行动计划”，将引进人才上升为娄底发展战略，有效解决人才瓶颈问题。铁腕整治阻碍娄底发展、影响娄底形象的人和事，坚决打掉各类黑恶势力及其“保护伞”，积极营造法治化、国际化、便民化的营商环境。

（四）全面深化改革创新，激发高质量发展活力

加大改革力度。持续推进供给侧结构性改革，遵循“巩固、增强、提升、畅通”的方针，进一步淘汰不安全落后产能。深化“放管服”改革，全面推进“双随机、一公开”“多证合一”“证照联办”和“证照分离”等改革，加快“互联网+政务服务”深度融合，做到“马上办、网上办、就近办、一次办”，努力实现“只跑一次”或“一次都不用跑”。大力推进国有企业、投融资平台、财税管理、农村集体产权等重点领域、关键环节改革，优化资源配置，增强微观主体活力。

扩大对外开放。坚定不移高举开放大旗，构建“面朝东南、多点支撑、全面开放”的新格局。围绕12条产业链，突出“三类500强”企业，紧盯沿海新一轮产业转移，继续推进产业链招商、小分队精准招商，确保内联引资增长15.5%以上、实际利用外资增长10.5%以上、进出口总额增长15.5%以上。持续实施“破零倍增”“娄企出海”计划，加快推进海关、铁路口岸、外贸服务平台建设，办好第十届“湘博会”。

强化创新驱动。立足薄板、高端装备制造、新能源、新材料、生物医药等重点领域，加强新一代信息技术的引进与应用。设立“双创”引导基金，加快建设一批产业创新联盟、国家级省级企业技术中心、重点实验室、工程技术研究中心，以及创新创业产业孵化示范基地、众创空间、创新工场，增强企业的科技创新源动力。深化产学研合作，加快科技成果转化。

（五）统筹城乡融合发展，拓展高质量发展空间

全面提升城镇品质。抓好新一轮城市总体规划编制，推动多规合一。继续加强城市“双修”，强力抓好控违拆违工作，开展违法建设专项整治，重点抓好中心城区道路通畅工程建设、旧城及老旧小区改造，加快推进供水供电供气管网设施、地下综合管廊建设、农贸市场分类提质改造，高标准推进高铁片区、涟钢片区、水洋片区、老街片区、华星路片区开发建设，打造城市新名片。加快县市城区提质扩容，推动要素快速集聚，完善城区服务功能，增强对周边发展的辐射力和带动力。进一步完善特色小城镇规划编制，加快高速公路沿线8个特色小城镇试点建设，培育一批工业强镇、商贸重镇、旅游名镇。加快推进龙琅高速建设，争取官新高速开工，进一步完善国、省干线公路网络。

大力推进乡村振兴。坚持农业农村优先发展，以“五个振兴”为目标，按照“盯住能人、发动群众、面向市场、科学奖补”的思路，推进“乡村振兴15条”落实落地。调优农业产业结构，鼓励土地集中流转，确保粮食总产160万吨、蔬菜产量158万吨。抓好“一县一特”主导产业培育，持续开展品牌强农行动，发展村级集体经济，打造以精细农业为特色的优质农副产品供应基地。促进农民专业合作社提档升级，力争全市新增农民专业合作社兴办企业500家。支持新化县创建全国农村一二三产业融合发展先导区，支持涟源市、双峰县创建省级农业标准化示范县（市），确保全年农产品加工销售收入、休闲农业企业经营收入分别增长12%、10%以上。实施“农村人居环境整治三年行动计划”，推进农村垃圾分类处置、厕所革命和村容村貌治理，抓好畜禽养殖场标准化改造，完成15个省定美丽乡村示范创建、6个市级美丽乡村建设任务。加强基层组织建设，选准配强村支两委班子，围绕解决“人、钱、窝”的问题，强化基本保障。做好科技特派员选送工作，支持市民下乡、能人回乡、娄商返乡、企业兴乡，鼓励社会各界人士投身乡村建设。

着力增强治理能力。巩固国家卫生城市创建成果，加快全国文明城市、国家森林城市创建。加快建设“智慧城市”，提高城市建设管理服务智能化、人性化水平。努力促进物业管理行业健康规范发展。发挥街道、社区作用，开展文明单位、文明村组、文明家庭创建，引导市民参与城市管理。做实“城市快警”“一村一辅警”，健全村民议事会制度，全面提升城市应急反应能力和农村自治能力，让全市人民更有归属感。

（六）着力保障改善民生，共享高质量发展成果

用心办好民生实事。继续推进湘江资江娄底流域截污和水体治理、易地扶贫搬迁、全科医生（助理全科医生）驻村全覆盖、农村垃圾分类处理、山塘清淤、化解“大班额”、背街小巷和老旧小区改造、自然村通水泥（沥青）路建设等8件实事。今年新增2件：中心城区停车场、停车位建设与管理，“公交都市”创建。

有效提升公共服务。落实教育优先发展战略，实施农村教师安心工程、农村学校饮水提标改造工程，深化新时代教师队伍建设改革，着力解决学前教育“入园难、入园贵”问题；促进教育均衡发展，中心城区新改扩建5所学校，启动4所学校建设，支持各县市新建或改扩建一批学校；积极发展面向市场的职业教育，支持娄底幼儿师范学校升专；推进集团化办学，做大做强本土名校，大力引进外地名校，打造一批知名教育品牌。全面推进“大众创业、万众创新”，积极开展“技能培训+创业培训”，推进人力资源市场建设，抓好高校毕业生、农民工、退役军人、贫困劳动力等重点群体就业创业，确保城镇新增就业3.6万人、新增农村劳动力转移就业3.5万人。深入推进分级诊疗，增强乡镇卫生机构服务能力，抓好村级卫生室标准化建设，全面提升全科医生驻村服务水平。加强公共文化服务，完成农村有线电视户户通惠民工程，市规划馆、科技馆、图书馆年内建成开馆。抓好殡葬领域突出问题整治，完成市殡仪馆就地扩容提质。

全面加强民生保障。完善社会保险制度，严厉打击骗取、套取社保基金违法行为。加快推进租购并举城市住房制度改革，全面推动以公租房、棚改房、安置房和农村危房改造为重点的保障性住房建设，全年新开工城镇棚户区住房改造5086套，完成农村危房改造3700套。大力发展健康养老产业，积极推进医养结合和公办养老服务机构建设，支持社会力量参与养老产业发展。做好社会救助兜底保障对象的调整、分类和归档工作，建立健全与经济社会发展相适应的低保标准调整机制，提高失能、半失能特困人员的集中供养率。推进福利慈善事业发展，落实残疾人“两项补贴”，完善农村留守儿童关爱保护体系。

切实维护社会稳定。以创建国家安全发展示范城市为抓手，严格落实安全生产责任，持续推进安全生产“十大战役”和“强执法防事故”行动，加强应急救援体系建设，坚决防范和遏制安全生产事故。落实“四个最严”要求，深入推进食用油整治等专项治理，抓好餐厨垃圾无害化处理，全面提升食品安全综合治理能力，维护人民群众“舌尖上的安全”。加快推进法治信访建设，打造网上阳光信访平台，深化信访工作“三无”创建。深入推进“平安娄底”“法治娄底”建设，更大力度开展“扫黑除恶”专项斗争，严厉打击严重刑事犯罪及黄赌毒黑拐骗等违法犯罪行为，进一步提升人民群众安全感。

加强国防动员后备力量建设，推进民兵编组和训练基地规范化建设。大力发展国防科技产业，加快打造省级以上军民融合产业园。做好退役军人安置保障工作。深入开展全国第二次污染源普查，做好第四次全国经济普查，抓好第二轮市志编纂。统筹抓好其它各项工作，多出特色亮点，多创娄底经验。

三、加强政府自身建设

坚持党的全面领导，树牢“四个意识”，坚定“四个自信”，做到“两个维护”，把全面从严治党、依法行政贯穿于政府自身建设始终，以服务促发展，以高效抓落实，以廉洁树形象。

大力解放思想。自觉践行新发展理念，对标先进地区和先进城市，着力破除陈旧落后的思想观念，摆脱传统的思维惯性和路径依赖，鼓励大胆实践、勇于创造，以观念的变革和思想的更新，助推高质量发展。

坚持干字当头。不务虚工、不唱高调，心无旁骛抓发展，真正把心思用到抓产业、优环境、强实体上来。切实改进作风，坚决反对形式主义、官僚主义，带头执行市委“十条规定”，重点整治慢作为、不作为，以“钉钉子”精神狠抓落实。关心关爱基层干部，树立真抓实干的导向，完善和落实激励约束、容错纠错机制，坚决落实“三个区分开来”，旗帜鲜明为担当者担当、为改革创新者撑腰。

严格依法行政。落实“三重一大”决策制度，做到依法、科学、民主决策。严格执行市人大及其常委会的决议决定，自觉接受人大法律监督与工作监督、政协民主监督和社会舆论监督，虚心接受人大评议，重大事项及时向人大报告、向政协通报，认真办理人大代表议案、建议和政协委员提案。深化行政执法体制改革，全面推进行政执法“三项制度”落实。加强政府诚信建设，说了就干，干就干成，做到言必信、行必果。

切实干净干事。把纪律和规矩挺在前面，贯彻执行中央八项规定及实施细则和省委“约法三章”，锲而不舍纠“四风”，严格落实“一岗双责”，坚持讲规矩、按制度办事，把廉洁从政贯穿到政府工作的各个环节，实现干部清正、政府清廉、政治清明。

各位代表！

使命呼唤担当，奋斗创造幸福。让我们高举习近平新时代中国特色社会主义思想伟大旗帜，在省委省政府和市委的坚强领导下，不忘初心、牢记使命，履职尽责、攻坚克难，为全面建成小康社会、加快建设富饶美丽健康祥和新娄底而努力奋斗！
